# 

**Published:** 1919-10-25

**Authors:** 


### The College of Nursing
### (Limited by Guarantee).

LIVERPOOL CENTRE.

MONDAY, OCTOBER 27, AT 7 P.M.—Miss Cowlin, Organising Secretary, will address a Members' Meeting in the lecture theatre of the Royal Infirmary on "The Future of the Nurse After Training." Admission will be free to members; non-members by ticket, price 6d., obtainable from Miss Golding, 11 Hargreaves Road, Sefton Park.

NOTTINGHAM CENTRE.

ON Thursday, October 16, the Nottingham Exchange Hall was well filled with an enthusiastic audience, who had come to support the Nation's Fund for Nurses and the College of Nursing. The Hon. Sir Arthur Stanley, C.B., M.V.O., G.B.E., spoke of the educational aims of the College, and of his hope that the Bill for the Registration of Nurses, to be brought forward by Dr. Addison, the Minister of Health, would be on the Statute Book by the end of this year. He mentioned that the abuse which had assailed the College throughout its three or four years of life had only stimulated its promoters to fresh efforts. Sir Arthur concluded by congratulating Nottingham nurses on the excellence of their Centre.

### A Matron's Resignation.

WITH reference to our note in *The Hospital* of October 18, Miss F. M. Tillson asks us to state that she is resigning from her present position as matron of the Gloucestershire Royal Infirmary, but is not retiring from institutional activity, as she intends to pursue her hospital work in other spheres.

